# Dr. James N. Ellis, Surgical Clinic at the Grady Hospital

**Published:** 1913-12

**Authors:** 


### DR. JAMES N. ELLIS, SURGICAL CLINIC AT THE GRADY HOSPITAL.

NOVEMBER 5TH, 1913.

Appendectomy: relief of ideal stasis by division of Ileo-Caecal Cicatricial Bands, Sequent to Typhoid Fever. The patient, a white male, about 30, was admitted to the hospital yesterday with a tenative diagnosis of appendicitis. His temperature was 99.4, pulse 100, respiration normal, leucocyte count 14,600; no nausea or vomiting; no palpable tumor, but with localized pain at McBurney's point, slight rigidity of the right rectus and pain in the right iliac region. Since typhoid fever, four years ago, his habitual good health has been interrupted by increasingly frequent attacks of abdominal pain, tenderness and discomfort, more marked in the right lower quadrant, disabling him for several days at a time.

This makes a fairly convincing picture of an acute exacerbation of chronic, recurrent appendicitis, but it was not conclusive or this man wound not have been operated on immediately. Remembering that some affections of the right ureter and kidney, such as unilateral nephritis, intermittent hydronephrosis, ureteral calculus, bacteriuria, etc., occasionally present a clinical picture closely simulating the one obtained so far, and the condition of the patient not seeming urgent, it was decided to defer operation pending a complete urinary analysis, to allow of some preliminary preparation and the opportunity for further continuous observation in the hospital.

This morning I find the patient's condition practically the same as when he entered the hospital yesterday. The urinary examination has been made and the report being negative in all essential particulars, the tentative pre-operative diagnosis is apparently confirmed, the operation is decided upon and anaesthesia, with ether by the drop method, will be induced.

As to when and how to operate in appendicitis, I have very decided views. The recognition of surgical intervention as the proper procedure in the treatment of appendicitis, coincides, in date, with the beginning of my professional life. Consequently, with the advent of my first case of appendicitis, a quarter of a century ago, a consideration of the then unsettled question of when to operate was immediately forced upon my attention I still remember my anxiety in the treatment of this first case and of those immediately succeeding it: of my indecision as to whether the time for surgical intervention had arrived, and of my subsequent unrest, when I left the patient, fearing I was prosrastinating and had allowed the pchychological surgical moment to pass.

Happily, for my present serenity of mind, and, I firmly believe, for the welfare of my patients, this period of perturbation, of uncertainty and of indecision on my part is long since a thing of the past. As a result of a continously increasing experience and observation, my views on this vexed question have become thoroughly crystallized into a conviction that practically every case of appendicitis demands immediate operation as soon as the nature of the trouble is unmistakably recognized and competent surgical aid can be obtained.

Appendicitis is a treacherous disease, and should be dealt with by the surgeon vigorously, aggressively, decisively and promptly. Delay, in supposedly convalescing patients, is fraught with danger and, often, with disaster. All of us who have had extended experience with appendicitis and who, in our days of doubt, have practiced expectant treatment have occasionally seen such apparently convalescing patients suddenly and unexpectedly develop a so-called general peritonitis, and die promptly, with or without surgical intervention. I have frequently operated on an apparently convalescing patient and found an appendix distended to its utmost capacity with pus and only the thin, serous investment intervening to protect the peritoneal cavity from a virulent, lethal infection.

The question with me now is not whether, or when, to operate, but what intervention shall I pratice in this particular case? This frequently can not be positively determined until the abdomen has been opened, and the exact pathological condition disclosed. My experience corroborates the teaching of Stuart McGuire that, so far as the surgeon is concerned, all cases of appendicitis may be resolved, for practical purposes, into four groups; namely: First, Chronic Appendicitis; Second, Acute

Appendicitis, without involvement of adjacent structures; Third, Acute Appendicitis, with circumscribed abscess; and Fourth, Appendicitis, with diffuse peritonitis.

The technique of operation in groups one and two are practically the same, and will be followed by me in the case before us,—the McBurney muscle-splitting, short incision being used to gain access to the cavity.

There is one method of dealing with the stump of the appendix, in these comparatively simple cases, which I wish to mention in order to condemn. I refer to the plan of burying the ligated, infected stump of the appendix by a superimposed pure string suture of linen or Paganstecker. The pocket thus made will necessarily become infected in a number of cases. While this does not kill, as the resulting abscess finally discharges into the caecum when the cat-gut ligature gives way, operation by this method is not infrequently responsible for a post-operative temperature of 100 to 101, observed as late as the seventh or eighth day after operation.

Operation in groups one and two is admittedly one of the safest and most satisfactory in surgery and is practically devoid of mortality.

In the third group, where the infection has extended to the neighboring structures with the formation of a circumscribed abscess, immediate operation is, in my opinion, just as imperative, whether the attack has lasted twelve hours or twelve days, but the procedure is varied according to the individual findings.

The abdominal incision is made over the most prominent part of the tumour and, if the obscess wall is found adhering to the underlying peritoneum, the pus is evacuated without entering the general cavity; the appendix removed, if readily accessible, but left, if difficult of access, and drainage established. If the abscess is not adherent to the anterior abdominal wall, the surrounding mass is carefully isolated by gauze pads, effectually protecting the general cavity, the pus carefully sought for and gently removed by sponging; the appendix found and removed if feasible; drainage instituted; the abdomen closed and the patient put in bed in the exaggerated Fowler position.

It is especially in the fourth group, namely, those with diffuse peritonitis, in which delay in operating and the institution of the Ochsner method of treatment is being quite extensively and enthusiastically advocated at the present time by both surgeons and physicians. But surely, if the surgeon restricts himself to making an outlet, or outlets from the peritoneal cavity for relief of tension and the escape of the accumulated purulent material, and provides for the temporary continuance of drainage, he has greatly facilitated the recovery of such a patient. It is after the establishment of such drainage that the inauguration of the Ochsner method of gastric lavage, prohibition of food by the mouth, rectal alimentation and proctolysis become life-saving measures and find their logical applicability.

The Ochsner method, combined with the continuous saline enema of Murphy, and the semi-erect posture of Fowler, applied as a post-operative procedure, is of incalculable value. As a substitute for imemdiate operation in appendicitis, it is deceitful and misleading, and, I believe, has proven a curse, by offering a seemingly safe course to timid patients and procrastinating, vacillating, temporizing, undecided surgeons.

Proceeding with the operation on the patient before us, we incise the peritoneum lifted between two haemostate, introduce the index finger to the inner side of the first part of the ascending colon, bring this to the surface, follow the longitudinal band over the caecum to the base of the appendix and deliver the organ without the abdominal cavity. Inspection and palpation discloses an apparently normal appendix, but with considerable turgescence of the vessels and some induration of the terminal one or two inches. There seems scarcely enough gross pathology here to warrant a positive diagnosis of appendicitis or to satisfactorily account for his symptoms. So, before removing this appendix, we will explore a little further.

Exhaustive study of the ileo-coscal region has recently culminated in a knowledge of many and varied pathological conditions in this vicinity which we formerly overlooked or misinterpreted, such as Jackson's veil, Lane's kink, caecum mobile, stenosis or incompetency of the ileocaecal valve, etc., presenting symptoms referable to the right lower quadrant of the abdomen suggestive of catarrhal or chronic appendicitis and, as a result of failure to recognize their pathological significance, many healthy, normal appendices were formerly erroneously removed in the expectation of relief, the essential lesion receiving no attention. Remembering, also, that this patient gives a history of having had typhoid fever some four years ago and that his trouble in this region is sequent to that attack, we naturally seek for an explanation of his symptoms in the neighborhood of the terminal twelve inches of the ileum; and here, just below the ileo-colic juncture, we find dense, cicatricial bands extending from the caput coli across the under surface of the mesentery to the small gut, drawing the ileum sharply downwards just before it terminates at the ileocaecal valve, and so angulating the gut as to form a mechanical obstruction closely simulating Lane's kink. This means functional interference, such as ileal stasis, and may very properly be construed as having materially contributed to the symptoms which this case presents and which, very probably, resulted from a local infection occurring by way of incomplete, pinhole perforations at the base of typhoid ulcers. We relieve this kink by severing these bands transversely, lifting the gut and then closing the hiatus in the peritoneum by catgut sutures, passing them somewhat after the manner used in the operation for incomplete harelip, a transverse incision with long individual closure. This, as you see, completely relieves the angulation and the consequent interference with the transit of the intestinal contents at this point.

We will now return to the appendix and remove it for, in of the fact that it is not, apparently grossly diseased, it is under suspicion, its excision does not materially add to the gravity of the operation and the microscope may reveal pathology enough to justify our suspicions. We ligate the meso-appendix, pass a purse string suture of No. 1 plain catgut around the base, sever the appendix between two Ferguson clamps, cauterize with carbolic followed by alcohol, invert the stump, tighten and tie the purse string and bury and reinforce this by a few stitches of No. 0 plain catgut. This method of dealing with the stump has served me so well that I have never been tempted, in order to save a few minutes' time, to modify my technique to the simpler methods now in vogue. Nevertheless, I heartily agree with Dr. Doyen, who once said to me, "You Americans have a saying that 'time is money;' I tell you that, in surgery, time is life. Do what you have to do well but do it quickly." The oft-quoted saying, "Get in quickly, get out quicker," does not apply to non-suppurative cases like this. Time is vital when infection and sepsis are profound, as in perforated, gangrenous, ruptured appendices with suppuration. The dilatory, time-consuming maneuvers of some surgeons under such circumstances is little short of criminal. In such cases we waste no time, but clamp the appendix at its base, ligate in the crease made by the clamp, amputate, drain and close the abdomen.

In the case before us, there is no indication or necessity for drainage and we close the small incision, in the usual way, with catgut throughout, the peritoneum with No. 0 plain, the aponeurosis of the external oblique with No. 1 chromic doubled and the skin with No. 0 plain.

---

(Report of Pathologist of The Carnegie Laboratory of Pathology and Bacteriology, Atlanta College of Physicians and Surgeons, Atlanta, Ga., Nov. 17, 1913.

Specimen: Appendix. This specimen consists of a cord-like structure measuring 10 cm. in length and 0.7 cm. in diameter. The center is canalized the diameter of which is 0.2 cm. The canal contains a brownish black pulpy substance not at all unlike fecal matter. On the outer surface are several rather dilated blood vessels filled with blood. The specimen is apparently made up of several coats, the outer of which is smooth and glistening. This coat covers a layer of muscle, internal to which two other layers, neither of which is very thick.

Microscopic Examination: There is not much evidence of swelling of the mucosa although the epitheleal cells of the crypts contain many small round cells which stain well and have small round deeply staining nuclei.

The muscle coats show no pathological change. In the peritoneal coat the vessels are engorged.

Diagnosis: There was present a mild chronic catarrhal inflammation involving the mucous membrance.

Respectfully submitted,

JOHN FUNKE.

---

Dr. Ellis will make a detailed report of the second case of the surgical treatment of cirrhosis of the liver with ascites, in a later number of The Journal-Record of Medicine.

---

Dr. F. K. Boland held a surgical clinic at Grady Hospital, Grady Hospital, at 10:30 Wednesday.

